# In Memoriam

**Published:** 1877-04

**Authors:** 


In Memoriam.—We learn from the *Southern Argus*, a newspaper published at Selma, Ala., of a loss the medical profession has sustained in the death of Dr. Samuel W. Vaughan, of Summerfield, Ala. He died on the 20th of December last, in the 77th year of his age. From youth he had been devoted to the science and practice of his profession, and with ability discharged the duties it imposed. Though for several years previous to, and during the war, he had somewhat relaxed from active practice, yet after the immense loss sustained by that destructive conflict, he again assumed the labor incident to a general practice.

We have known of Dr. Vaughan for many years, and have had the pleasure of an intimate acquaintance with two of his sons, who graduated in the Medical College of this city, with distinction. While it is unreasonable to expect the services of one so ripe in years to be prolonged to the extent we might desire, a vein of sadness and regret passes through us at the loss of an honorable, upright citizen and useful member of the medical profession. Peace to his remains!

